# Repeated photon and C-ion irradiations *in vivo* have different impact on alteration of tumor characteristics

**DOI:** 10.1038/s41598-018-19422-x

**Published:** 2018-01-23

**Authors:** Katsutoshi Sato, Nobuhiro Nitta, Ichio Aoki, Takashi Imai, Takashi Shimokawa

**Affiliations:** 1 Cancer Metastasis Research Team, Advanced Radiation Biology Research Program, Research Center for Charged Particle Therapy, National Institute of Radiological Sciences, QST, 4-9-1 Anagawa, Inage-ku, Chiba, 263-8555 Japan; 2 Clinical Genetic Oncology, Cancer Institute Hospital, Japanese Foundation for Cancer Research, 3-8-31 Koto-Ku, Tokyo, 135-8550 Japan; 3 Department of Molecular Imaging and Theranostics, and Group of Quantum-state Controlled MRI, National Institute of Radiological Sciences, QST, 4-9-1 Anagawa, Inage-ku, Chiba, 263-8555 Japan; 4 National Institute of Radiological Sciences, QST, 4-9-1 Anagawa, Inage-ku, Chiba, 263-8555 Japan

## Abstract

Precise characterization of tumor recurrence and regrowth after radiotherapy are important for prognostic understanding of the therapeutic effect. Here, we established a novel *in vivo* mouse model for evaluating the characteristics of regrown tumor after repeated photon and carbon ion (C-ion) irradiations. The results showed that tumor growth rate, lung metastasis, shortening of the survival of the tumor-bearing mice, and tumor microvessel formation were promoted 2- to 3-fold, and expression of angiogenic and metastatic genes increased 1.5- to 15-fold in regrown tumors after repeated photon irradiations, whereas repeated C-ion irradiations did not alter these characteristics. Interestingly, both repeated photon and C-ion irradiations did not generate radioresistance, which is generally acquired for *in vitro* treatment. Our results demonstrated that the repetition of photon, and not C-ion, irradiations *in vivo* alter the characteristics of the regrown tumor, making it more aggressive without acquisition of radioresistance.

## Introduction

With the development of current irradiation techniques, it is now possible to deliver higher radiation dose into local tumor. Stereotactic radiotherapy (SRT) is a typical technique and it can be used to irradiate local tumors with more than 10 Gy of photons in each fractionation^1^. SRT is now applied to various cancers such as brain tumor^2^, lung^3^, liver^4^, and prostate cancer^5^. Especially in early stage lung cancer, the therapeutic outcome is approximately 70% for 5 year overall survival rate^3^. Moreover, particle radiotherapies such as carbon ion (C-ion) radiotherapy are also significantly effective for tumor control. The favorable outcome of C-ion radiotherapy is based on the evidence of higher relative biological effectiveness (RBE) and excellent dose distribution. The RBE, the ratio of the radiation dose of the reference radiation that is required to induce a given effect to the dose of the radiation of interest that is required to produce the same effect, of C-ions is more than two fold greater than photons. In addition, the ionization of the C-ion reaches a maximum at the end of the beam path, and then steeply drops sparing tissues beyond the tumor location. This is known as the Bragg peak, and the peak can be precisely determined to occur at the tumor site for an ideal dose distribution. These properties are advantageous to photon resistant tumors such as osteosarcoma^6^ and melanoma^7^. Furthermore, late stage cervical cancer has been recently treated with C-ion radiotherapy, with clinical results showing a higher local control rate with few complications in the surrounding organs^8,9^.

On the other hand, local recurrence and treatment failure has also been observed in some cases. In the case of early stage lung cancer, the local recurrences were observed in 14^3^ and 23%^10^ of patients who are treated with hypofractionated SRT and C-ion radiotherapy respectively. In addition, it was reported that 25 and 33% of patients demonstrate locoregional failure after C-ion radiotherapy of squamous cell carcinoma and adenocarcinoma in uterine cervix, respectively^11^. In general, the recurrent tumor and regrown tumor after radiotherapy is rarely re-treated with conventional broad beam radiotherapy because the surrounding normal tissues cannot tolerate additional irradiation. Moreover, the regrown tumor might acquire more aggressive and radioresistant characteristics after repeated irradiations. Although clear evidence of this notion has not been demonstrated in clinical and *in vivo* studies, it is supported by published *in vitro* data that used repetitive photon irradiations that promote the metastatic potential via various phenomena such as enrichment of cancer stem cell fractions^12^ and epithelial to mesenchymal transition^13^. Moreover, we also previously elucidated that repetitive photon irradiations *in vitro* conferred significant photon and C-ion resistance in cancer cells^14,15^. Since these phenotypic changes definitely impair the patient’s prognosis and we could not find any published research with *in vivo* models of regrown tumor after repeated photon or C-ion irradiations, the impact of repeated irradiations *in vivo* on tumor characteristics including metastatic potential and radiosensitivity need to be understood.

In this study, we assessed whether the characteristics such as tumor growth, metastatic potential, and radiosensitivity are changed in regrown tumors by establishing a novel *in vivo* regrown tumor models after repeated photon or C-ion irradiations. We report the difference in alteration of these characteristics between regrown tumor after repeated photon or C-ion irradiations *in vivo*.

## Results

### Repeated photon irradiations *in vivo*, but not repeated C-ion irradiations, significantly promoted the tumor growth potential

**Figure 1:**
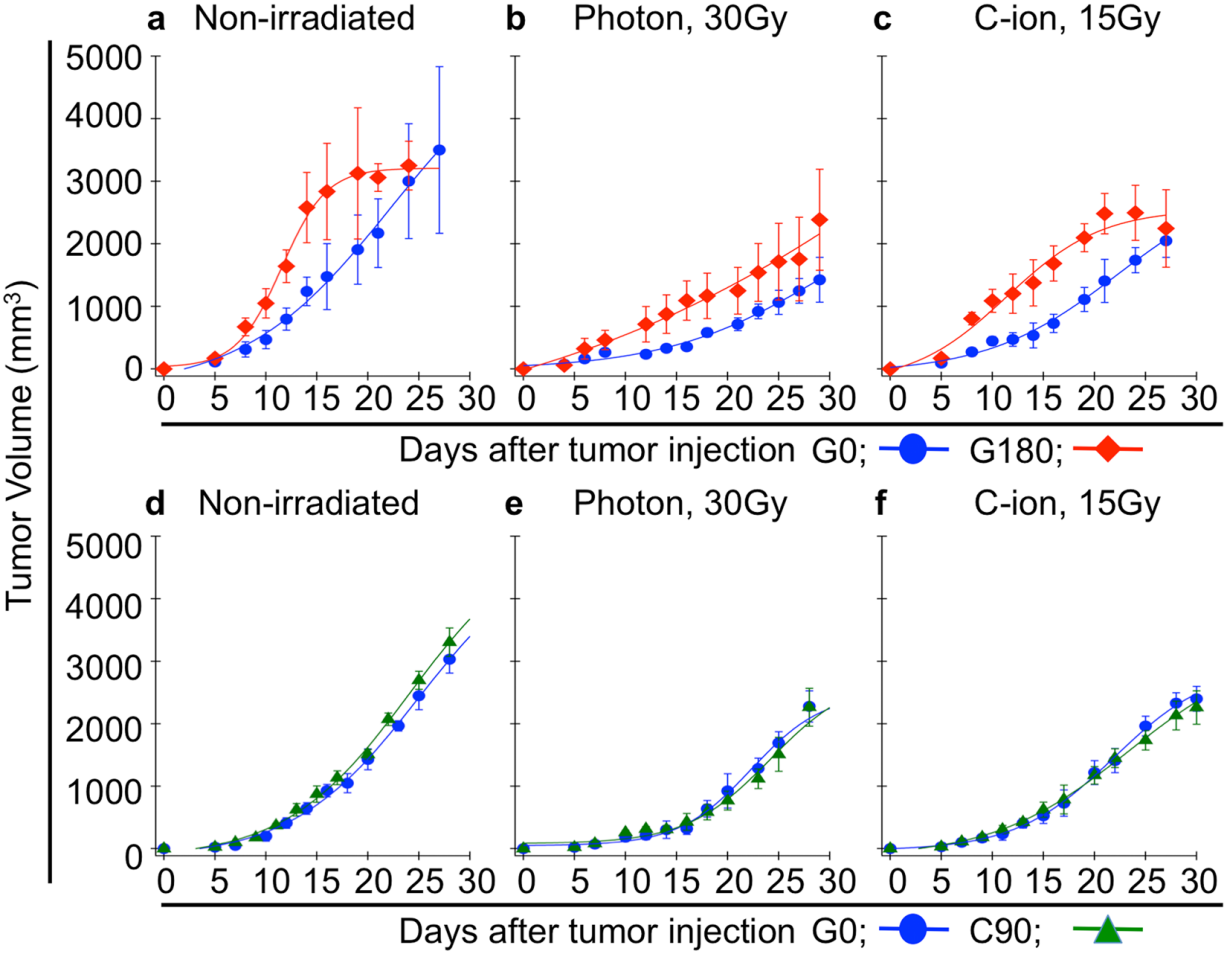
Tumor growth potential. (**a**–**c**) and (**d**–**f**) show change in tumor volume of G180 and C90 tumor in comparison with that of G0 tumor, respectively. The data of non-irradiated, 30 Gy of photon irradiated, and 15 Gy of C-ion irradiated condition are shown in (**a**,**d**), (**b**,**e**), and (**c**,**f**), respectively. The blue, red and green color are respectively indicates the value of G0, G180 and C90 tumor. The data shown by mean ± standard deviation.

To assess whether the influence of repeated irradiations on tumor characteristics *in vivo* differ depending on the type of irradiation, we first established regrown tumors after repeated photon and C-ion irradiations. It is confirmed that NR-S1 tumor are radioresistant mouse squamous cell carcinoma cells arising from buccal mucosa and are able to easily form metastatic nodules on the lung surface^16^. In this study, the NR-S1 tumors were inoculated into the right hind leg of C3H/He mouse, after which the tumors were irradiated respectively with 30 Gy or 15 Gy of photon or C-ion irradiation. Since a previous study^17^ that the RBE value of NR-S1 cells was approximately 2, these dosages of photon and C-ion were able to be regarded as very approximately biologically equivalent. In fact, the tumor growth (Fig. 1) and the growth rate of NR-S1 tumor after 30 Gy of photon irradiation (Fig. 1b and e) were approximately same as that after 15 Gy of C-ion irradiation (Fig. 1c and f). The irradiated NR-S1 tumors were subsequently transplanted into intact C3H/He mice after 2 weeks of irradiation, and then the regrown tumors were irradiated again 2 weeks after the transplantation. This procedure was repeated 6 times, and the 180 Gy photon-irradiated, 90 Gy C-ion-irradiated, and non-irradiated tumors were established as G180, C90, and G0 tumors, respectively (Supplemental Figure 1).

**Figure 2:**
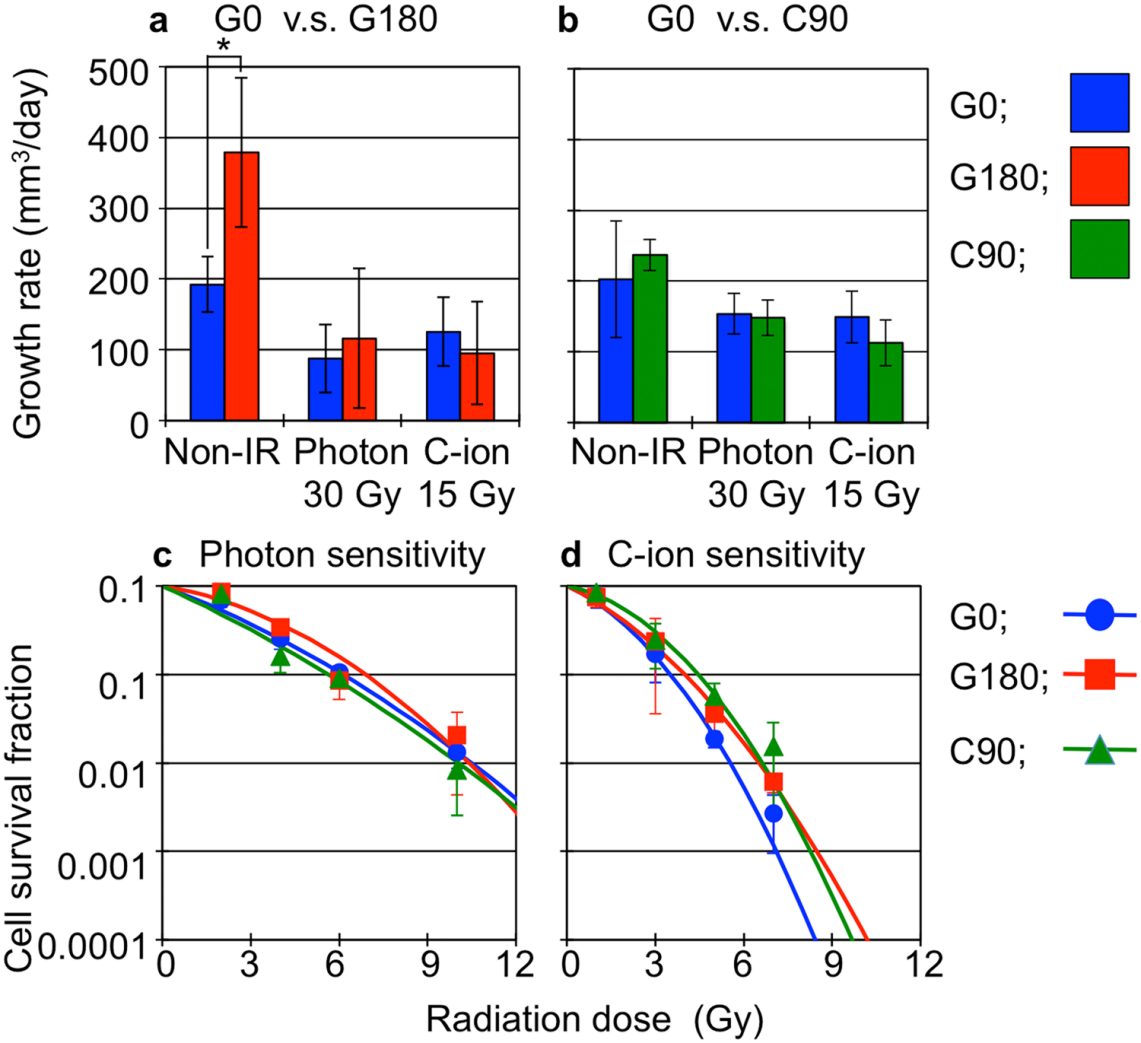
Tumor response to photon and C-ion irradiation, and radiosensitivity of each tumor cells. (**a**) and (**b**) respectively shows tumor growth rate of G180 and C90 tumor compared with that of G0 tumors. The values were calculated with the linear part of tumor growth showing in Fig. 2. The blue, red and green bars respectively indicate the values for G0, G180 and C90 tumors. The data shown by mean ± standard deviation. (**c**) and (**d**) show the cell survival after photon C-ion irradiation, respectively. The cell survival of G0, G180, and C90 cells are respectively shown by blue, red and green line. The values and error bars are respectively mean and standard deviation.

To evaluate the difference in tumor growth between G0, G180, and C90 tumor, we first measured the tumor volume (Fig. 1). In the non-irradiated condition, the tumor growth of G180 tumors was clearly promoted compared with that of G0 tumors (Fig. 1a). The growth rate, which is calculated with the linear part of growth curves, was 192.5 and 378.9 mm^3^/day for G0 and G180 tumors, respectively (Fig. 2a). We next assessed the effect of the X-ray and C-ion irradiation on tumor growth. Although the tumor volume of G180 tumors was larger than that of G0 tumors even after photon or C-ion irradiation (Fig. 1b and c), the tumor growth rate of G180 tumors was approximately the same as that of G0 tumors after photon or C-ion irradiation. The tumor growth rate of G0 tumors after photon or C-ion irradiation were 87.4 or 125.6 mm^3^/day and the growth rate of G180 tumors were 116.1 or 95.2 mm^3^/day (Fig. 2a), respectively. On the other hand, tumor growth rate of C90 tumors in non-irradiated condition, after photon or C-ion irradiations, were not significantly different from that of G0 tumors (Fig. 1d–f, and Fig. 2b). The tumor growth rate of C90 tumors in the non-irradiated condition, photon, or C-ion irradiation were 237, 148.1, or 112.5 mm^3^/day, respectively.

These results suggested that the photon and C-ion sensitivities of G180 and C90 tumors were not altered compared with that of G0 tumor. To evaluate this, we established *in vitro* cell lines of G0, G180 and C90 tumors, and measured the photon and C-ion sensitivity by means of the colony formation assay. The results showed that the photon and C-ion sensitivity in G180 and C90 tumor cells was approximately the same as that of G0 tumor cells (Fig. 2c and d).

These results showed that the repeated photon irradiations *in vivo*, but not the repeated C-ion irradiations, significantly promoted the tumor growth potential, In addition, the repeated photon or C-ion irradiations *in vivo* did not alter the photon or C-ion sensitivity (Supplemental Tables 1 and 2). This means that the radioresistance *in vivo* was not induced by repeated photon or C-ion irradiations.

### Repeated photon, and not C-ion *in vivo* irradiations promoted the metastatic potential of the regrown tumor

**Figure 3:**
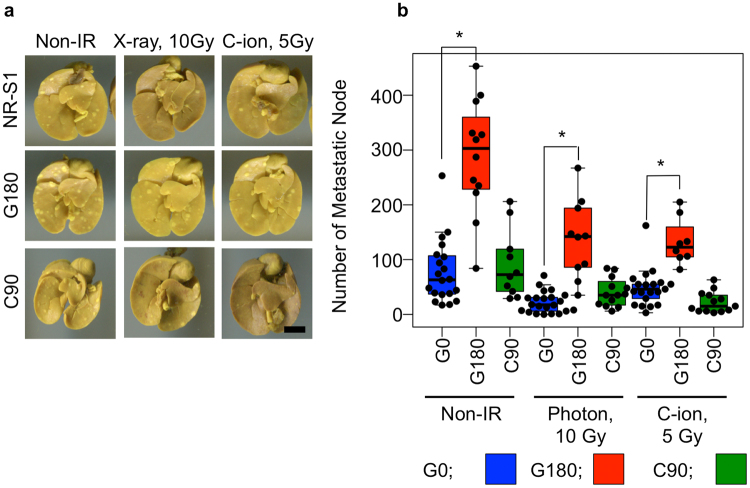
Metastatic potential. (**a**) shows the basal part of the lung with metastatic nodule. The scale bar showing in the right bottom panel indicates 5 mm, and this scale is able to be applied in all photographs. (**b**) shows summary of the values. The plots indicate the number of metastatic nodule of each tumor-bearing mouse. The top and bottom side of the boxes are 25 and 75 percentile. The upper and lower whiskers are maximum and minimum value. If the maximum number of the nodules is more than the1.5-times interquartile range, the value is regarded as the outliers. The horizontal bar in each box is median value.

G180 tumors had significant higher growth potential than G0 and C90 tumors in non-irradiated conditions (Figs 1, 2a and b). Next, we hypothesized that the G180 tumors also have higher metastatic potentials than G0 and C90 tumors. To evaluate the metastatic potential of each tumor, we counted the metastatic nodules on the lung surface in each tumor inoculated mouse. As we expected, the number of metastatic nodules of G180 tumor-bearing mice was significantly higher than that of G0 and C90 tumors in the non-irradiated, photon and C-ion irradiated conditions (Fig. 3). In the non-irradiated condition, the mean number of metastatic nodules for G0, G180, and C90 tumors were 79.7, 254, and 91.5, respectively. After 10 Gy of photon irradiation, the numbers for G0, G180 and C90 tumors were decreased to 22.0, 137.1, and 39.3, and the respective suppression rates were 28, 54, and 43% of that in the non-irradiated condition. Likewise, C-ion irradiation suppressed the lung metastasis of each tumor. The number of metastatic nodes for G0, G180, and C90 were decreased to 37.4, 132.6, and 22.3 after 5 Gy of C-ion irradiation, respectively. The values were 47, 52, and 24% of that in non-irradiated condition, respectively.

These results clearly indicated that only the repeated photon, and not C-ion *in vivo* irradiations promoted the metastatic potential of the regrown tumor. However, the metastatic potential of each tumor was well suppressed by additional photon or C-ion irradiation although the number of metastatic nodules in G180 tumor bearing mice was still higher than that in C90 and G0 tumors after additional photon or C-ion irradiation.

### Regrown tumor after repeated photon irradiations impaired survival of tumor-bearing mice

**Figure 4:**
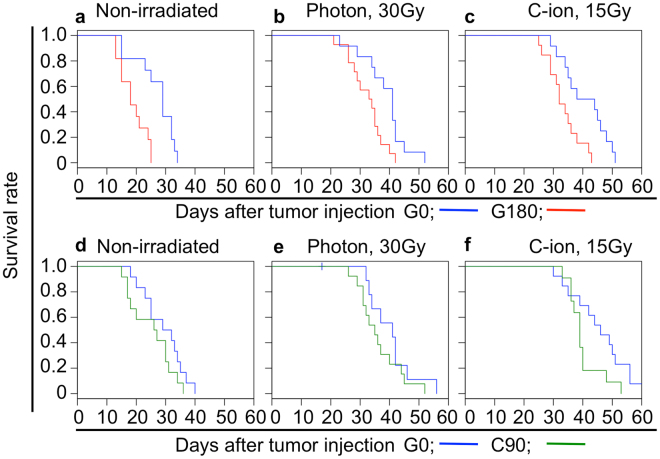
Survival of tumor-bearing mice. (**a**–**c**) and (**d**–**f**) show survival of G180 and C90 tumor-bearing mice in comparison with that of G0 tumor-bearing mice, respectively. These were independently acquired in different experiment. The data of non-irradiated, 30 Gy of photon irradiated, and 15 Gy of C-ion irradiated condition are shown in (**a**,**d**), (**b**,**e**), and (**c**,**f**), respectively. The blue, red and green color are respectively indicates the value of G0, G180 and C90 tumor. The results of the statistical analysis are summarized in Table 1.

The enhancement of the tumor growth (Figs 1, 2a and b) and metastatic potential (Fig. 3) suggests that the G180 tumors may shorten the survival time of tumor bearing mice compared with G0 and C90 tumors. To verify this assumption, we measured the survival time of G0, G180, and C90 tumor-bearing mice. As expected, the G180 tumor remarkably shortened the survival time of the tumor bearing mice (Fig. 4 and Table 1). The median survival time of G180 and G0 tumor bearing mice was 18 and 29 days, respectively. The 30 Gy of photon or 15 Gy of C-ion irradiation significantly extended the survival time of G180 tumor-bearing mice compared with non-irradiated conditions. However, the survival time was significantly shorter than the survival time of G0 tumor bearing mice after photon or C-ion irradiation (Fig. 4b,c and Table 1). The median survival time of G180 tumor-bearing mice was 33.5 or 32.0 days after photon or C-ion irradiation, while they were 34.8 or 41 days, respectively, for G0 tumor-bearing mice. On the other hand, the survival time of C90 tumor-bearing mice was approximately the same as that of G0 tumor-bearing mice. In addition, no statistical difference was detected between the survival time of photon or C-ion irradiation of the C90 and G0 tumor-bearing mice (Fig. 4d–f and Table 1).

**Table 1:** Survival periods of G0, G180, and C90 tumor-bearing mice.

	Comparison between G0 and G180						Comparison between G0 and C90					
	Non-irradiated		Photon, 30 Gy		C-ion, 15 Gy		Non-irradiated		Photon, 30 Gy		C-ion, 15 Gy	
	G0	G180	G0	G180	G0	G180	G0	C90	G0	C90	G0	C90
Number of mice	11	11	12	14	12	13	12	12	14	13	13	11
Median (day) (Min.-Max.)	29.0 (15–34)	18.0 (13–25)	34.8 (12–42)	33.5 (21–42)	41.0 (29–51)	32.0 (25–43)	30.5 (18–40)	26.5 (15–36)	33.5 (17–56)	35.0 (26–52)	46.0 (30–61)	39.0 (33–40)
*p*-value	—	0.003	—	0.015	—	0.009	—	0.159	—	0.251	—	0.061

These results clearly indicated that the regrown tumor after repeated photon irradiations became aggressive. On the other hand, the repeated C-ion irradiation did not influence the aggressiveness of the regrown tumor.

### Tumor vasculature in G180 tumor was significantly enhanced

**Figure 5:**
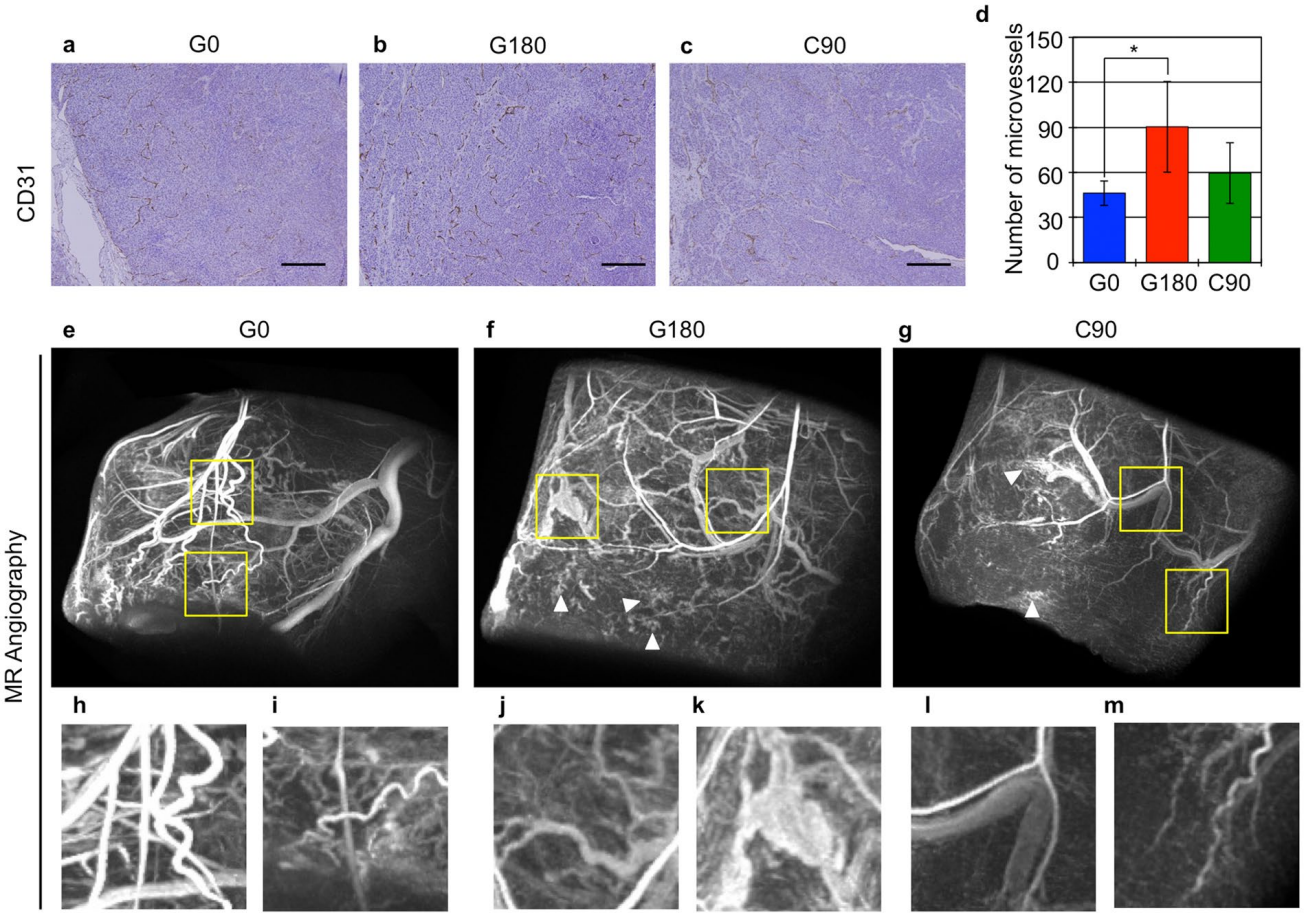
Evaluation of tumor microvessels using immunohistochemical staining and magnetic resonance angiography. (**a**–**c**) show the tissue section immunostaining with anti-CD31 antibody. The scale bar shows 250 µm. (**d**) shows the number of CD31 positive microvessels in each tumor. The values indicated the mean ± standard deviation. The asterisk means statistical significant compared with the value of G0 tumor. (**e**–**m**) Shows 3D MR micro-angiography of the indicated tumor. (**h**,**i**), (**j**,**k**) and (**l**,**m**) were indicated that the magnified images of the area enclosing yellow square in (**e**), (**f**) and (**g**), respectively.

Tumor aggressiveness might be associated with enhancement of tumor angiogenesis^18^. Therefore, we counted the CD31 positive cells, which represent the vascular endothelial cells, to measure the number of microvessels in each tumor. The results showed that the number of CD31 positive cells was significantly increased in the G180 tumors compared with G0 and C90 tumors, but the number in C90 tumors was approximately the same as that in G0 tumors (Fig. 5a–c, and Supplemental Figure 2). These data indicate that there were more microvessels in G180 tumors compared with G0 and C90 tumors, and suggest that the tumor vasculature of G180 tumors is different from that of G0 and C90 tumors. To further evaluate the difference in tumor vessels between each tumor, magnetic resonance angiography was performed with a PEGylated liposomal MRI contrast agent^19^. The results showed that overall tracking of tumor vessels in G0 tumor (Fig. 5e) was relatively traceable from its stem (Fig. 5h) to peripheral (Fig. 5i). While, overall tracking of tumor vessels in G180 tumor is difficult to be traced (Fig. 5f), indicating that the vascular network in G180 tumor was more complex. In G180 tumor, the shapes of peripheral tumor vessels were not smooth (Fig. 5j), and the tumor vessels were irregularly enlarged compared with that in G0 and C90 tumor (Fig. 5k). In addition, the blood flow was diffused at the tumor periphery (Fig. 5f, arrowhead). On the other hand, the overall tracking (Fig. 5g), stem (Fig. 5l) and peripheral shape (Fig. 5m) of tumor vessels in C90 tumor were somewhat similar to G0 tumor. Whereas, the blood flow at the tumor periphery was also diffused (Fig. 5g. arrowhead).

These data indicated that the repeated photon irradiations altered the angiogenic potential of the regrown tumor and the tumor vessels in the regrown tumor became unstable. On the other hand, repeated C-ion irradiations had less influence on the angiogenic and vessel formation of the regrown tumor.

### Gene expression associated with promotion of tumor vasculature and metastatic potential was increased in G180 tumor

**Figure 6:**
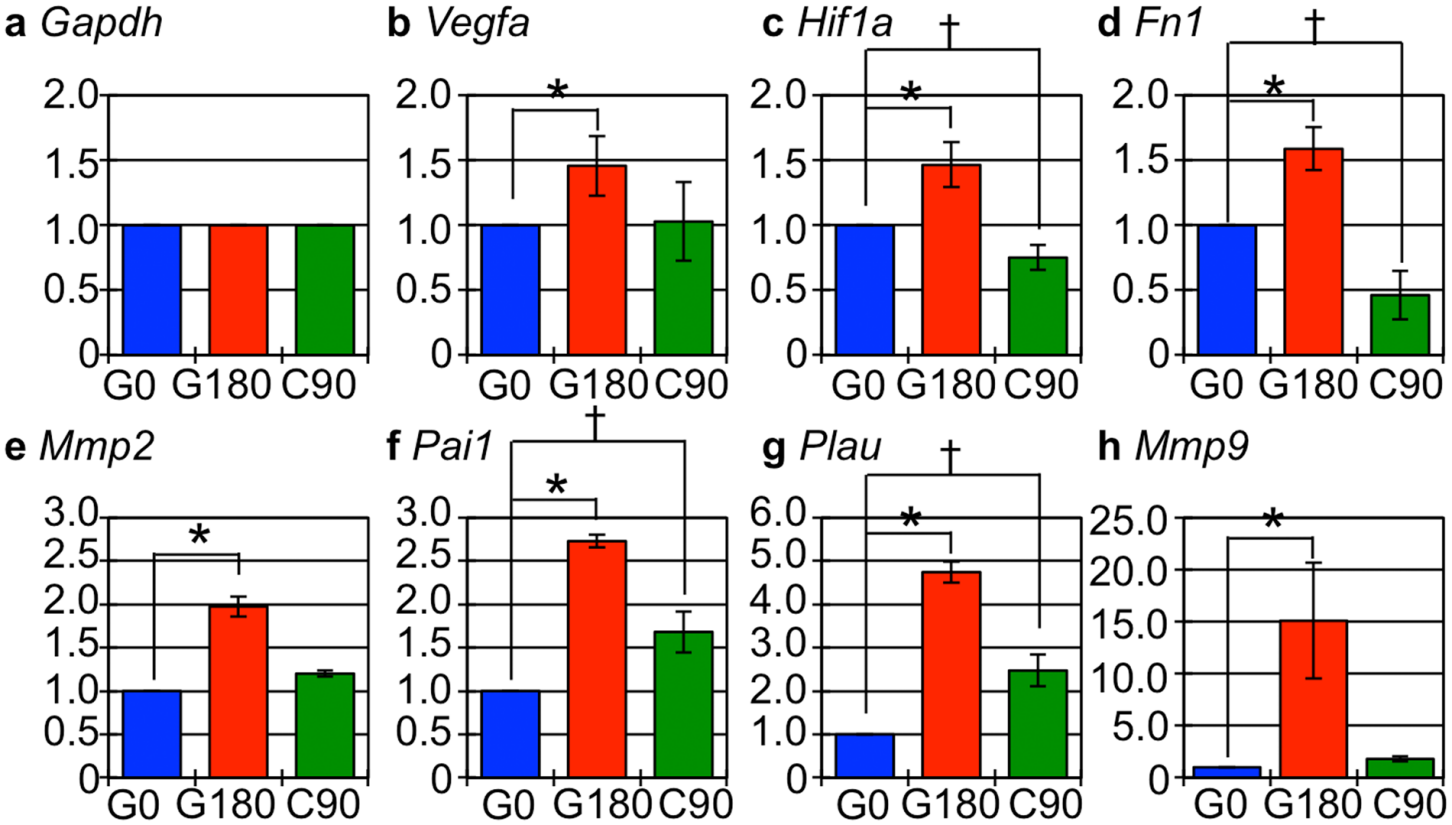
Angiogenesis and metastasis related gene expression. (**a**–**h**) show the fold-changes in the indicated genes. The blue, red and green boxes showed the genes in G0, G180 and C90 tumor, respectively. The values indicated the mean ± standard deviation. The asterisk and dagger means statistical significant compared with the value of G0 tumor.

The tumor growth (Figs 1 and 2) and metastatic potential (Fig. 3) of G180 tumor was significantly increased and the survival period of G180 tumor-bearing mice was markedly shortened compared with that of C90 and G0 tumor (Fig. 4). In addition, G180 tumor contained numerous microvessels (Fig. 5). These results suggest that expression of the genes that are related to angiogenesis and metastasis were promoted in G180 tumors. To assess this, we measured the expression of typical angiogenesis and metastasis related genes, *Vegfa* (Vascular endothelial growth factor A), *Hif1a* (Hypoxia inducible factor 1 A), *Fn1* (Fibronectin), *Mmp2* (Matrix metalloproteinase 2), *Pai1* (Plasminogen activator inhibitor), *Plau* (Urokinase plasminogen activator), and *Mmp9* ((Matrix metalloproteinase 9), which also are regulated by HIF1α, by quantitative PCR analysis. The results showed that expression of these genes were significantly increased in G180 tumors compared with that of G0 tumor. For C90 tumor, the expression of only two genes, namely *Pai1* and *Plau*, were significantly increased, while *Hif1a* and *Fn1* genes were significantly decreased compared with that of G0 tumor (Fig. 6).

These results indicated that the repeated photon irradiations upregulated typical angiogenesis and metastasis related genes, whereas the influence of the repeated C-ion irradiations on the expression of these genes was slight. This indicates that the type of irradiation has different impact on the expression of tumor related genes.

## Discussion

We showed that the repeated photon irradiations promoted aggressiveness represented by the enhancement of tumor growth, metastatic potential, and increase in the number of tumor vessels in the regrown tumor (Figs 1–5). In line with these results, the expression of genes associated with angiogenesis and tumor invasion also significantly increased in G180 tumor (Fig. 6). On the other hand, the repeated C-ion irradiations, the single fractioned dose of which is biologically equivalent to that of the photon irradiation, did not contribute to the aggressiveness and only slightly influenced the gene expression. These results indicated that the impact of repeated photon irradiations on phenotypic change and gene expression, which are closely related with tumor aggressiveness, is different from that of C-ion irradiation.

Regarding the characteristics of recurrent tumor after radiotherapy, some groups have reported that chromosomal aberration^20,21^ and promotion of growth potential represented by the increase in KI67 positive cancer cells^22^ were found in clinical specimens of recurrent glioblastoma, cervical cancer, and meningioma after radiotherapy. However, there is no clear evidence demonstrating that the recurrent tumor acquired the different tumor characteristics including more aggressive nature compared with that prior to the initial radiotherapy. In *in vitro* study, the differences in angiogenic and metastatic response between photon and C-ion irradiation have been previously reported by some groups. Kamilah F. *et al*. showed that the photon irradiation, but not C-ion irradiation, increased tumor microvessels in A549 tumors, correspondingly upregulating stromal cell derived factor 1 (SDF-1), one of the angiogenic factors^23^. Likewise, photon-induced angiogenesis in human lung adenocarcinoma cell line A549 was also reported by Girdhani S. *et al*. Their study demonstrated the angiogenesis via VEGF secretion and HIF1α expression was promoted by a sublethal dose of photon irradiation, while the same dose of high energy proton irradiation could be suppressed^24^. They mentioned that the results complemented the results of C-ion irradiation that was reported by other groups. In addition, Ogata T. *et al*. showed that the low dose of photon irradiation increased the gene expression and the protein activity of MMP2 and MMP9, and they promoted the metastatic potential of human fibrosarcoma cell line HT1080 and mouse osteosarcoma cell line LM8 cells, while the biological equivalent dose of C-ion irradiation suppressed the metastasis^25^. Similar results also observed in A549 cells^26^ and some human glioma cell lines^27^. Moreover, Suetens A. *et al*. indicated that C-ion irradiation could well suppress the expression of genes related to cell motility compared with photon irradiation^28^. Although these data support our results, they do not explain the difference in the characteristic change of regrown tumor.

As for *in vivo* studies, Camphausen K. *et al*. showed eradication of primary tumor on tumor-bearing mice by a high dose of photon irradiation, the total doses of which are ranging from 30 to 50 Gy, significantly promoted the formation of metastatic nodules on the lung surface^29^. Although it supported that photon irradiation might contribute to increase the probability of metastasis *in vivo*, their data did not indicate the changes in characteristics of the irradiated tumor itself. Therefore, to our knowledge, the acquired characteristics of regrown tumor have never been reported previously. Contrary to these studies, we established *in vivo* regrown tumor models to investigate the influence of the repeated photon or C-ion irradiations on the phenotypic changes, and consequently demonstrated for the first time that the regrown tumor after repeated photon irradiations, but not after C-ion irradiations, acquired significant aggressiveness that is associated with enhancement of angiogenic and metastatic potential. Therefore, our results are novel and notable evidence showing that *in vivo* characteristics, especially aggressiveness, of regrown tumor after repeated irradiations were clearly different between photon and C-ion irradiations.

The reason why the photon irradiations, but not C-ion irradiations, promote aniogenesis and metastasis in the irradiated cancer cells is poorly understood. However, the difference in HIF1α response between X-ray and C-ion irradiation might be speculated as one of the possible reasons. HIF1α is widely known as a transcription factor for various genes including *VEGF*, *PAI1*, *uPA*, *FN1*, and *MMP2*. The gene expression, protein stability, and transcription activity of HIF1α were increased not only in hypoxic condition^30^ but also after X-ray irradiation^31,32^. Moreover, the microenvironment such as hypoxic areas might also be associated with the difference in tumor aggressiveness between photon and C-ion irradiation. Harada, *et al*. explained the mechanisms of tumor recurrence after photon irradiation by evaluating *in vivo* dynamics of tumor cells in the hypoxic area. They showed that the tumor cells in severe hypoxic areas preferentially survived after 25 Gy of photon irradiation, and subsequently these tumor cells migrated toward tumor vessels. This migration was suppressed by HIF1 inhibitor YC-1, indicating that the migration of the surviving tumor cells after photon irradiation is HIF1α dependent^33^. On the other hand, it is known that C-ion irradiation is able to effectively suppress HIF1α activity^34^ and its expression^35^, and equally killed the tumor cells regardless of the hypoxic or normoxic status^35–39^. Our results also showed that the gene expression of *Hif1a* and its downstream target genes were increased in G180 tumor. On the contrary, the expression levels of the target genes, apart from *Plau* and *Pai1*, in C90 tumors were approximately the same or lower than that of G0 tumors (Fig. 6). Therefore, our results showed that the repeated photon irradiations *in vivo* contribute to the acquisition of tumor aggressiveness corresponding with upregulation of Hif1α signaling, whereas the repeated C-ion irradiations *in vivo* do not change the tumor aggressiveness, and only slightly affected the Hif1α signaling. Although the reason for the increase in the expression of *Pai1* and *Plau* genes in C90 tumors must be cautiously assessed in further studies since the Pai1 and Plau proteins are correlated with metastatic potential of cancer cells^40,41^, treatment outcome of radiotherapy^42^, and poor prognosis of cancer patients^43,44^, our results also mean that C-ion irradiation might be superior to photon irradiation in regard to preventing acquisition of tumor aggressiveness.

Here, we should interpret that whether cancer stem cell (CSC) fractions were enriched in the G180 tumor. It is reported that CSC fractions, which were represented by tumor cells with surface marker such as SOX2 (Sex determining region Y-box 2)^45^, OCT3/4 (Octamer binding transcription factor 3/4)^45^, CD133 (Cluster of differentiation 133)^12,46–48^, CD44^46–49^, and EpCAM (Epithelial cell adhesion molecule)^47,48^ could be increased by repetition of photon irradiations, while C-ion irradiation could effectively kill the CSC fractions in the tumor^49^. Since these studies were performed both *in vitro*^12,45–49^ and *in vivo*^49^ and the total radiation doses that were used in their studies ranged from 2 to 2278 Gy^12,45–49^, the experimental conditions of these studies covered with that dose range. It suggests the increase of CSC fractions was likely to have occurred also in G180 tumor, but not in C90 tumor. In fact, CSC fractions in G180 tumor was higher than that in C90 and G0 tumor, because the *in vitro* cultured cells that were isolated from G180 tumor contained a lot of EpCAM positive cells and have significantly higher sphere formation capacity, which is prominent characteristics of CSC, compared with that from C90 and G0 tumor (Supplemental Figures 4 and 5). However, the tumor growth rate after photon and C-ion irradiation was approximately same as each tumor (Fig. 2a,b), and the survival fractions of G0, G180, and C90 cells were approximately same (Fig. 2c,d). It means that the CSC fractions in G180 tumor contributed less to promote tumor aggressiveness and to acquire the radioresistance although repeated photon irradiations likely enriched the CSC fractions. To conclude the association with CSC in our regrown tumor models, the number of CSC fractions in G0, G180 and C90 *in vivo* condition were assessed by further study.

Another interesting finding in our study was that the radioresistance in the regrown tumor could not be induced by either the repeated photon or C-ion irradiations *in vivo*. Previously, we have demonstrated the repeated photon irradiations altered the irradiated cells into radioresistant cells *in vitro*. In this study, the NR-S1 cells, which are the same as G0 tumors, were irradiated with 10 Gy of photon six times equaling a total dose of 60 Gy to establish NR-S1-X60 (X60) cells. The X60 cells acquired not only photon resistance but also C-ion resistance compared with NR-S1 cells^14,15^. On the contrary, our results *in vivo* showed that X-ray and C-ion resistance in G180 and C90 tumors were not induced by repeated photon or C-ion irradiations (Fig. 1). This reason might be associated with the difference in microenvironment surrounding the irradiated cells *in vitro* and *in vivo. In vitro* systems are composed of just one type of cell, and the culture space is spatially limited by the culture dish. This means that factors produced by irradiated cells such as growth factors^50^, cytokines^51^, cell free DNA^52^, and miRNA^53^ may continuously affect the cells unless these factors are removed or inactivated. The cells continue to be exposed to these factors even after the irradiation, and this may eventually contribute to adapt the cells to the additional irradiation. In contrast, the microenvironment in *in vivo* studies is definitely complex. It is composed of many types of cells including cancer cells, stromal cells, and immune cells. Moreover, effective range of the irradiated cell derived factors *in vivo* may be larger, and the duration may be shorter than that *in vitro* because these factors are diffused by the tumor vasculature. In this condition, the irradiation itself and the irradiated cell derived factors may affect not only the cancer cells of interest but also other host cells such as stromal and immune cells, and spatial and temporal effects of these factors may be heterogeneously changed along with time in both the implanted tumor and the host (Supplemental Figure 5). If the microenvironment such as tumor vessel formation has potent impact (Fig. 5) for the survival of the cancer cell after repeated irradiations rather than acquisition of radioresistance in cancer cells, the radioresistant cancer cells like X60 cells *in vitro*^14,15^ are hardly generated *in vivo*. Although some subjects such as the ratio of cellular population within the tumor, phenotypic changes of cancer cells itself, and conformation of the generality using multiple cell lines have to be studied to elucidate the detailed mechanisms of selection by repeated irradiations *in vivo*, our results indicated that influences of the repeated photon or C-ion irradiations on radioresistance induction differ between *in vitro* and *in vivo* circumstances.

In conclusion, we showed that the repeated photon irradiations *in vivo* promote the tumor aggressiveness that is represented by enhancement of tumor growth, angiogenesis, and metastasis, and impaired survival of the tumor bearing mice. On the other hand, the repeated C-ion irradiations *in vivo* only slightly influenced these characteristics of the regrown tumor. In addition, we showed that the radioresistance in the regrown tumor was not induced by either repeated photon or C-ion irradiations *in vivo*. To our knowledge, there is no other study showing that the tumor characteristics *in vivo* after repeated photon irradiations were different from that after repeated C-ion irradiations, and that the radioresistance was not induced by *in vivo* repeated irradiations. Although further studies using multiple cell lines are required to confirm whether these phenomena are a general event for all tumors or not, we demonstrated the evidence that the repeated photon irradiations *in vivo* promote tumor aggressiveness without radioresistance, while the repeated C-ion irradiations *in vivo* has less effect on such tumor characteristics.

## Methods

### Cell lines

Mouse squamous cell carcinoma cell line NR-S1, a kind gift from Dr. Koichi Ando (Medicine and Biology Division, Gunma University Heavy ion medical center), was used. The NR-S1 cells were established from spontaneously occurring tumor in buccal mucosa of female C3Hf/He mouse^16^. The cells were maintained in DMEM (Wako, Osaka, Japan) containing 10% fetal bovine serum (Sigma-Aldrich) and 0.1% penicillin/streptomycin (Gibco^®^, Carlsbad, CA).

### Animal experiment

Seven week old female C3H/He mice were purchased from Japan SLC, Inc. (Shizuoka, Japan). Before beginning the experiments, the mice were bred to allow 2 time to habituate for 1 week. The animal experiments in this study were approved by the National Institute of Radiological Sciences Institutional Animal Care and Use Committee, and all experiments were performed in accordance with relevant guidelines and regulations (protocol No. 12-2005-3, 13-2017).

### Irradiations

PANTAK (Shimazu, Kyoto, Japan) and ^137^Cs source (RSG-50, Toshiba, Tokyo, Japan) were used for X-ray and γ-ray irradiation, respectively. The tube current, voltage, focus-surface distance (FSD), dose rate for the X-ray irradiation was 20 mA, 200 kVp, 1.0 Gy/min, and 55 cm, respectively. The dose rate and FSD for the γ-ray irradiation was 1.0 Gy/min. and 30 cm, respectively. The fluctuation of radiation dose within the radiation field was less than 10% for both X-ray and γ-ray. The mouse body other than the tumor on right hind leg was shielded from both X-ray and γ-ray exposure by using originally designed lead collimator, thickness of which is 5 cm. To deliver planning radiation dose into the tumor, radiation dose of X-ray was monitored with ionization chamber (C-110, Applied Engineering Inc, Tokyo, Japan.) during the irradiation experiment. For γ-ray irradiation, the radiation dose was controlled by means of adjusting the irradiation time with a time switch that is installed on RSG-50 irradiator. The radiation dose of γ-ray that would be exposed to the tumor was confirmed with radiophotoluminescent glass dosimeter (GD-302M, AGC Techno Glass Co., LTD. Shizuoka, Japan) in the actual experimental setup position, and the radioactivity of ^137^Cs was corrected for decay since installation. All instruments for measuring the radiation dose of X-ray or γ-ray were constantly calibrated at National Institute of Advanced Industrial Science and Technology, the institute of which is Primary Standard Dosimetry Laboratory in Japan.

C-ion irradiation was performed at the Heavy Ion Medical Accelerator in Chiba (HIMAC) of the National Institute of Radiological Sciences, Japan. The energy and dose rate of C-ion were 290 MeV/nucleon and 5 Gy/min., respectively. For the C-ion irradiation, 10 cm spread out Bragg peak (SOBP) in depth was adopted and the tumor was irradiated at the center of the SOBP. The dose-averaged linear energy transfer (LET) was approximately 55 keV/µm^54^, and the field uniformity of C-ion irradiation was within ± 2.5%^55^. The mouse body other than tumor was shielded with brass collimator that is installed on the HIMAC irradiation gantry. The irradiation experiments were performed at room temperature.

### Establishment of regrown tumor models after repeated X-ray or C-ion irradiations

To establish *in vivo* regrown tumor models, we repeatedly irradiated NR-S1 tumors with γ-ray or C-ion (Supplemental Figure 1). The NR-S1 tumors on the right hind leg of C3H/He mice were irradiated with 30 or 15 Gy of γ-ray or C-ion, respectively. Two weeks after irradiation, the tumor was excised, digested into single cell, and then the cells were inoculated again into the intact mice. This protocol was repeated six times, and thus the NR-S1 tumors were eventually irradiated with 180 Gy or 90 Gy of γ-ray or C-ion beam, respectively. After completing the sequence of protocols, the non-irradiated NR-S1, the NR-S1 tumors irradiated with 180 Gy in total of γ-ray and 90 Gy in total of C-ion were established as “G0”, “G180”, and “C90” tumors, respectively. We defined G180 and C90 tumors as the regrown tumor after γ-ray and C-ion irradiations, respectively. The details are described in Supplemental information.

### Colony formation assay

To measure the X-ray and C-ion sensitivity of the G0, G180, and C90 tumor cell itself, we performed the colony forming assay. G0, G180, and C90 tumor were excised, and digested into single cells following the above methods. The cell suspension of each tumor was seeded to cell culture dishes, and maintained in 10% fetal bovine serum (Sigma-Aldrich) containing, DMEM (WAKO, Osaka, Japan), and then *in vitro* G0, G180, and C90 cells were established. The X-ray or C-ion irradiation, and the colony formation assay using *in vitr*o G0, G180 and C90 cells was performed according to our previous report^14,15^.

### Preparation of tumor-bearing mice

Three weeks before experiments, the stored G0, G180, and C90 tumors were thawed, and 2.0 × 10^6^ cells were injected into the right hind leg of C3H/He mice. Then the mice were euthanized, each tumor was excised, digested into single cell, and 1.0 × 10^6^ cells were injected to the right hind leg of C3H/He mice for the measurement of tumor growth, lung metastasis, and survival of tumor-bearing mice.

### Tumor growth

After 1 week of tumor injection, the G0, G180, and C90 tumors were irradiated with 30 Gy of X-ray or 15 Gy of C-ion. After the irradiation, the tumor diameter and thickness was measured with a caliper for 30 days. The tumor volume was calculated using the following equation:

$${\rm{T}}.{\rm{V}}.=(\pi /6)\times {\rm{L}}\times {\rm{S}}\times {\rm{T}}$$

where T.V., L, S, and T are tumor volume, tumor diameter in long, short axis, and height of tumor, respectively.

### Lung metastasis

After 1 week of tumor injection, the G0, G180, and C90 tumors were irradiated with 10 Gy of X-ray or 5 Gy of C-ion beam. After 2 weeks of irradiation, the mice were euthanized with 1 ml of 5 mg/ml Somnopentyl (Kyoritsu Seiyaku Corp.) per mouse, and the lung was excised. The lung was fixed with Bouin solution, and the metastatic nodes on the lung surface were macroscopically counted with a magnifying glass.

### Survival of tumor-bearing mice

After 1 week of tumor injection, the G0, G180, and C90 were locally irradiated with 30 Gy of X-ray or 15 Gy of C-ion beam. The survivability of the mice was measured for 60 days. The survival curves were calculated with Kaplan-Meier method.

### Immunohistochemical staining

The paraffin embedded tumor was stained with anti-CD31 antibody (AB28364, Abcam, Cambridge, England) for detection of vascular endothelial cells in tumor. The details are described in Supplemental information.

### MR angiography

To obtain the magnetic resonance (MR) angiography, the 91 µg/100 µL/mouse of PEGylated liposomal contrast agent attached with Gd-DOTA chelate (Gadolisome^®^, DS Pharma Biomedical Co., Ltd. Japan)^19^ was intravenously injected into the mice, and then the contrast enhanced MR images were acquired using 7.0T-MRI (Biospec AVANCE-III System, Burker Biospin, Switzerland) with a cryogenic radio-frequency coil (2-ch phased array, transmission and reception, Burker Biospin) using Gradient Echo (FLASH) sequence (see Supplemental information in detail). The images were reconstructed using ParaVision software (Burker Biospin) and analyzed by OsiriX (ver. 4.1.2, 64 bit, Pixmeo, Switzerland).

### Gene expression analysis

The excised tumor was minced, enzymatically digested, and isolated to single cells. The dead cells, red blood cells, and leukocytes were removed from the single cell solution, and the viable tumor cells were enriched by means of the methods described in Supplemental information. The gene expression of *Gapdh*, *Vegfa*, *Hif1a*, *Fn1*, *Mmp2*, *Pai1*, *Plau*, and *Mmp9* were analyzed by quantitative polymerase chain reaction (qPCR). The primers used in this study are summarized in Supplemental Table 1, and the detailed methods are described in Supplemental information.

### Statistical analysis

Statistical differences in tumor growth curves and survival curves were tested by analysis of variance and log-rank test, respectively. The comparison of results for each tumor was assessed by Dunnett’s test.

## Electronic supplementary material

Supplemental information
