# Supplementary material for: Repeated photon and C-ion irradiations in vivo have different impact on alteration of tumor characteristics
Source: Sci Rep. 2018 Jan 23;8:1458. doi: 10.1038/s41598-018-19422-x (PMC5780469; doi:10.1038/s41598-018-19422-x)

Title; Repeated photon and C-ion irradiations *in vivo* have different impact on alteration of tumor characteristics

Authors; Katsutoshi Sato[1,2], Nobuhiro Nitta[3], Ichio Aoki[3], Takashi Imai[4], Takashi Shimokawa*, [1]

Affiliations of authors;

1. Cancer Metastasis Research Team, Advanced Radiation Biology Research Program, Research Center for Charged Particle Therapy, National Institute of Radiological Sciences, QST, 4-9-1 Anagawa, Inage-ku, Chiba, 263-8555, Japan.
2. Clinical Genetic Oncology, Cancer Institute Hospital, Japanese Foundation for Cancer Research, 3-8-31 Koto-Ku, Tokyo, 135-8550, Japan.
3. Department of Molecular Imaging and Theranostics, National Institute of Radiological Sciences, QST, 4-9-1 Anagawa, Inage-ku, Chiba, 263-8555, Japan.
4. National Institute of Radiological Sciences, QST, 4-9-1 Anagawa, Inage-ku, Chiba, 263-8555, Japan.

*. Corresponding author

Contact information of corresponding author; Takashi Shiomkawa,

Cancer Metastasis Research Team, Advanced Radiation Biology Research Program, Research Center for Charged Particle Therapy, National Institute of Radiological Sciences, QST, 4-9-1 Anagawa, Inage-ku, Chiba, 263-8555, Japan.

E-mail; shimokawa.takashi@qst.go.jp, Tel; +81-43-206-4048, Fax; +81-43-206-6267

Supplemental information

Establishment of regrown tumor model after repeated X-ray or C-ion irradiations

To establish in vivo regrown tumor models, we repeatedly irradiated NR-S1 tumor with γ-ray or C-ion (Supplemental fig. 1). The NR-S1 cells were harvested with PBS(-) (Nissui Pharmaceutical Co. Ltd.) and Trypsin-EDTA (Gibco), and then 2×10^6 cells were injected into the right hind leg of C3H/He mice. Three weeks after the injection, the mice were euthanized with 1 ml of 5 mg/ml Somnopentyl (Kyoritsu Seiyaku Corp., Tokyo, Japan) per mouse and the tumors were aseptically excised and minced. The minced tumors were incubated with digestion solution composed of PBS (-) (Nissui Pharmaceutical Co., LTD.) containing 0.2 % trypsin (BD Biosciences, San Jose, CA), 0.02 % pancreatin (Sigma-Aldrich, St Louis, MO), and 0.1 % of DNase I (Roche Diagnostics, Basel, Switzerland), for 20 min. at 35 ˚C. After the digestion, the cell suspension was repeatedly pumped more than 10 times with syringe, filtrated with 200 μm square sterilized mesh, and then PBS (-) containing 10 % FBS (Nissui Pharmaceutical Co., LTD.) was added to inhibit the digestion. The cell suspension was filtrated with 70 μm square sterilized mesh, centrifuged with 500 g for 10 min., and 2×10^6 of the tumor cells were injected into the left hind led of intact C3H/He mice. Two weeks after injection, the mice were anesthetized with 100 μL of 5 mg/ml Somnopentyl

(Kyoritsu Seiyaku Corp., Tokyo, Japan) per 10 g of body weight, and the NR-S1 tumors were irradiated with 30 or 15 Gy of γ-ray or C-ion, respectively. Two weeks after irradiation, the irradiated tumor was excised, digested into single cell, and then 2×10^6 cells were injected again into the intact mice. This protocol was repeated six times, and thus the NR-S1 tumors were eventually irradiated with 180 Gy or 90 Gy of γ-ray or C-ion beam, respectively. After completing the sequence of protocols, the tumor-bearing mice were bred for 4 weeks to regrow the irradiated tumors, then the tumors were excised, digested into single cells, and the cells were stored with CellBanker solution (Takara Bio Inc., Shiga, Japan) at -80 ˚C.

The non-irradiated NR-S1, the NR-S1 tumors which were irradiated with 180 Gy in total of γ-ray and 90 Gy in total of C-ion were named as “G0”, “G180”, and “C90” tumors, respectively. We defined G180 and C90 tumors as the regrown tumor after γ-ray and C-ion irradiations, respectively.

Colony formation assay

To measure the X-ray and C-ion sensitivity of the tumor cell itself, we primarily cultured the G0, G180, C90 tumors, and then the colony formation assay was performed. Each tumor was excised, digested into single cells following the above methods. The cell suspensions of G0, G180, and C90 tumors were seeded to cell culture dishes, and maintained

in DMEM (WAKO, Osaka, Japan) containing 10 % fetal bovine serum (Sigma-Aldrich). The X-ray or C-ion irradiation, and the following colony formation assay were performed as described in our previous report[15].

Immunohistochemical staining

The excised tumors were fixed with 10 % buffered formalin (WAKO) for 1 day, and then the tumors were embedded into paraffin with Tissue-Tek® VIP® (Sakura Finetek Japan Co., Ltd., Tokyo, Japan). The paraffin embedded tissues were sliced at 3 μm thickness, the tissue sections were deparaffinized with xylene, dehydrated with ethanol, and antigen retrieval was performed with 10mM citrate buffer using autoclave. After that the sections were washed twice with PBS(-), blocked with 5 % non-fat dry milk for 1 hour at room temperature, and incubated with Anti-CD31 antibody (AB28364, Abcam, Cambridge, England) overnight at 4 ˚C. Then the sections were washed twice with PBS (-), and incubated with Biotinylated anti-rabbit IgG (H+L) (Vector laboratories, Inc., Burlingame, CA) for 1 hour at room temperature. The tissue sections were stained with Vestastain® elite ABC kit (Vector Laboratories, Inc.) according to the manufacture's protocol.

MR angiography

The tumor bearing mice were continuously anesthetized during the magnetic resonance (MR) imaging by inhalation of 2 % isoflurane in 90 % air and 10 % O_2. After the anesthesia, the mice were fixed on Cryogenic RF coil (2ch, phased array, Burker Biospin, Switzerland) by the mouse fixture cradle. To obtain MR angiography, 91 μg/100 μL/mouse of PEGylated liposomal contrast agent conjugating with Gd-DOTA polyamidoamine dendron[19] was intravenously injected into the mice, and then the contrast-enhanced images were acquired by means of time of flight method with 7.0T-MRI (Biospec AVANCE-III System, Burker Biospin) using following parameters: Gradient Echo (FLASH sequence), TR = 15 ms, TE = 2.9 ms, number of acquisition = 3, flip angle = 20°, field of view = 17.0 × 12.8 × 12.8 mm^3, matrix = 340 × 256 × 256, spatial resolution = 50 × 50 × 50 μm^3 and scan time = 36 min 51 s.

Gene expression analysis

Each tumor was harvested as described above, and the minced tumor was incubated with RCB buffer (Biolegend, San Diego, CA) for 3 min. to hemolyze red blood cells in the tumor. After centrifuging and aspirating the supernatant, the tumor pellets were resuspended with DMEM (Nissui) to mitigate the low osmotic effect of RBC buffer. To remove the cellular debris, density gradient centrifugation using Percoll® solution (GE Healthcare UK

Ltd.) were performed. The Percoll stock solution was diluted to 7 % and 3 % with PBS(-). The 7 %, 3 % Percoll solution and the tumor cell suspension were slowly layered in the 50 ml centrifugation tube as the bottom, middle, and upper layer respectively, and then the tube was centrifuged at 2,000 rpm for 20 min. The upper layer was removed by aspiration, and the tumor pellet at the bottom was harvested. To remove the dead cells and the leukocyte fractions from the tumor pellet, Dead cell removal kit (Miltenyi Biotec GmbH, Bergisch Gladbach, Germany) and CD45 microbead (Miltenyi Biotec GmbH) were used, respectively. Subsequently, the living tumor cells without leukocyte was collected.

The RNA from the living tumor cells was harvested with ReliaPrep (Promoga, Madison, WI) following the manufacture's protocol. The RNA was reversely transcribed into cDNA using PrimeScript® RT reagent Kit (Takara Bio Inc., Shiga, Japan). Quantitative PCR was performed using THUNDERBIRD SYBR qPCR Mix (TOYOBO Co., Ltd., Aichi, Japan). The primers and annealing temperatures used in this study are listed in Supplemental table. 1. The fold-changes of the gene expressions were calculated by ΔΔCT method. The cycle threshold (CT) value of *Gapdh* for G0 tumor was adopted as the control of for the calculation of ΔΔCT.

Supplemental figure legends

Supplemental fig. 1.

Procedures for establishment of regrown tumor.

Supplemental fig. 2.

Magnified immunohistochemical staining tissue sections with anti-CD31 antibody. (**a**-**c**) shows respective images in Fig. 5(**a**-**c**) respectively. The scale shows 100 μm.

Supplemental fig. 3.

EpCAM and CD44 expression of G0, G180 and C90 cells. Each tumor was excised and cultured *in vitro* conditions. After more than 2 weeks of culture, percentages of EpCAM and CD44 positive cells were assessed by flow cytometery. Each cells were washed twice with PBS(-), and harvested by 0.05 w/v % Trypsin- 0.53 mmol/L EDTA solution (Wako), and 1.0 x10^6 cells were incubated with EpCAM (Alexa Flour®647 anti-mouse CD326, 118208, BioLegend Inc., San Diego, CA, USA) and CD44 antibody (FITC anti-mouse/human CD44,

103006, BioLegend Inc.) for 1 hour at 4°C. After that the cells were washed twice with PBS(-), and then the fluorescence intensity was measured by flow cytometer Gallios (Beckman Coulter, Brea, CA, USA). (**a**-**b**) shows the dot plot of EpCAM (vertical axis) and CD44 intensity (horizontal axis) for in G0 (**a**), G180 (**b**) and C90 (**c**) cells, respectively. LL, UL, LR, and UP respectively mean lower left, upper left, lower right, and upper right compartment. (**d**) shows the vertical profile of (**a-c**). Blue, red, and green show the intensity profile of G0, G180, and C90 cells, respectively. (**e**) shows that the percentages of each compartment shown in (**a-c**). Blue, red, and green boxes indicate G0, G180, and C90 cells, respectively. Asterisk showed statistically difference compared with the value of G0 cells ($p < 0.05$, Dunnett's test). (**f**) show the mean fluorescent intensity of EpCAM in the region that is indicated by two headed arrow in (**d**). Asterisk shows statistically difference compared with the value of G0 cells ($p < 0.05$, Dunnett's test).

Supplemental fig. 4.

Sphere formation potential of G0, G180 and C90 cells. Each tumor was excised and cultured *in vitro* conditions. After more than 2 weeks of culture, 500 cells were seeded into non-adherent culture dish (EZ-BindShut® II, AGC Techno Glass Co. Ltd., Shizuoka, Japan.), and were cultured for 2 weeks with 10 % FBS (Sigma-Aldrich), 0.8% methyl cellulose

(Wako), and 0.03 % gellum gam (Wako) containing DMEM (Nissui Pharmaceutical Co. Ltd., Tokyo, Japan). After that, resultant spheroids were stained as black with MTT solution (Wako). The culture dishes were imaged with flatbed scanner (GT-S630, Seiko Epson Corp., Nagano, Japan.), and counted the spheroid. Sphere formation efficiency was calculated by dividing number of resultant spheroid by number of seeded cells. (**a**) shows the typical image of resultant spheroid in G0, G180 and C90 cells. (**b**) shows that magnified images of single spheroid shown in (**a**). These images are acquired with same magnification of microscope. (**c**) shows that the sphere formation efficiency. Blue, red and green indicate the data of G0, G180 and C90. Asterisk shows statistically difference compared with the value of G0 cells ($p < 0.05$, Dunnett's test). Dagger means that the difference was not statistically significant.

Supplemental fig. 5.

Difference in tumor characteristics during the establishment. (**a**) shows changes in tumor growth rate at the indicated total dose of photon (red) and C-ion (green) irradiation compared to unirradiated controls (blue). The values and errors bar show the mean and standard deviation, respectively. Asterisk showed the statistical difference compared with the value at 0 Gy ($p < 0.05$, Dunnett's test). These data showed that the tumor growth rate of photon irradiated tumor was begun to promote at the time that the total dose was reached to more than 120 Gy of photon

irradiation. On the other hand, the tumor growth of C-ion irradiated tumor was approximately same as that of non-irradiated tumor. (**b-d**) show the DNA contents of G0, G180, and C90 cells. (**e**) indicates the percentage of cells in each phase. Blue, red, and green show respectively that the percentages of G0, G180, and C90 cells. Asterisk shows the statistical difference compared to the value at 0 Gy ($p < 0.05$, Dunnett's test). The DNA contents analysis showed that there are a lot of polyploid cells in G180 and C90 cells, while G0 cells contained a few polyploid cells. These results suggested that the cancer cell itself in the tumor might be gradually changed the characteristics during repeated photon irradiations, especially at more than 120 Gy.

Supplemental table 1. List of primers for quantitative PCR analysis

Genes	Forward primer	Reverse primer	Annealing temperature set in this study (˚C)
Gapdh	GGTGTGAACGGATTTGGCCGTATTG	CCGTTGAATTTGCCGTGAGTGGAGT	60
Vegfa	GCACCCACGACAGAAGGAGAGCAGA	CAGGGTCTCAATCGGACGGCAGTAG	60
Hif1a	TGACGGCGACATGGTTTACATTTCTGA	TCCCTTTTCTCACTGGGCCATTTCTG	60
Fn1	CTGGAGGCAAACCCTGACACTGGAG	CTGCCCGTTCGTGGGGGTAGTAGTT	60
Mmp2	GTTGCCCCCTGATGTCCAGCAAGTA	GGAGTCTGCGATGAGCTTAGGGAAACC	60
Pai1	CCAGCGCCTCTTCCACAAGTCTGAT	CACAACGTCATACTCGAGCCCATCG	60
Plau	CCAGGGGGAGCACTGTGAGATA	CAGGTCTGTGGGCATTGTAGGG	60
Mmp9	CCAGAGCGTCATTCGCGTGGATAAG	TGGTCCACCTTGTTCACCTCATTTTGG	60

Supplemental table 2. Difference in growth potential and radiosensitivity between G0, G180, and C90 tumor..

	Tumor growth rate (mm^3/day) (Mean ± Standard deviation)						Radiation sensitivity			
	Comparison between G0 and G180			Comparison between G0 and C90			Radiation dose at 10 % of survival fractions (Gy)			
	G0	G180	*p*-value (t-test)	G0	C90	*p*-value (t-test)	G0	G180	C90	*p*-value (Anova)
Non-IR	192.5 ± 39.4	378.9±105.3	<0.01	202.7±82.2	237.0±21.9	0.52	-	-	-	-
X-ray	87.4 ± 48.1	116.1±98.5	0.57	154.0±28.6	148.1±25.2	0.74	5.5±0.5	5.6±0.6	5.7±0.4	0.87
C-ion	125.6 ± 48.8	95.2±72.5	0.48	149.2±36.6	112.5±32.5	0.10	3.9±1.9	3.6±0.7	3.7±1.1	0.97

Supplemental table 3. Summary of differences in characteristics of G180 and C90 compared with that of G0 tumor (* shows statistically difference).

	Fold-increased in G180 tumor			Fold-increased in C90 tumor		
	Non-IR	Photon	C-ion	Non-IR	Photon	C-ion
Tumor growth rate	x2.0* Enhanced ($p = 0.008$)	x1.3 Same	x0.8 Same	x1.2 Same	x1.0 Same	x0.8 Same
Radiation sensitivity at D10 dose	-	x1.0 Same	x1.0 Same	-	x1.0 Same	x1.0 Same
Metastatic potential	x3.7* Enhanced ($p < 0.001$)	x6.3* Enhanced ($p < 0.001$)	x3.5* Enhanced ($p < 0.001$)	x1.2 Same	x1.8 Same	x0.6 Same
Survival of tumor bearing mice	x0.7 * Shortened ($p = 0.003$)	x0.6* Shortened ($p = 0.015$)	x0.8* Shortened ($p = 0.009$)	x0.9 Same	x1.0 Same	x0.8 Same
Tumor microvessel formation	x2.0* Increased ($p = 0.002$)	-	-	x1.3 Same	-	-

Supplemental figure 1, Shimokawa *et al.*

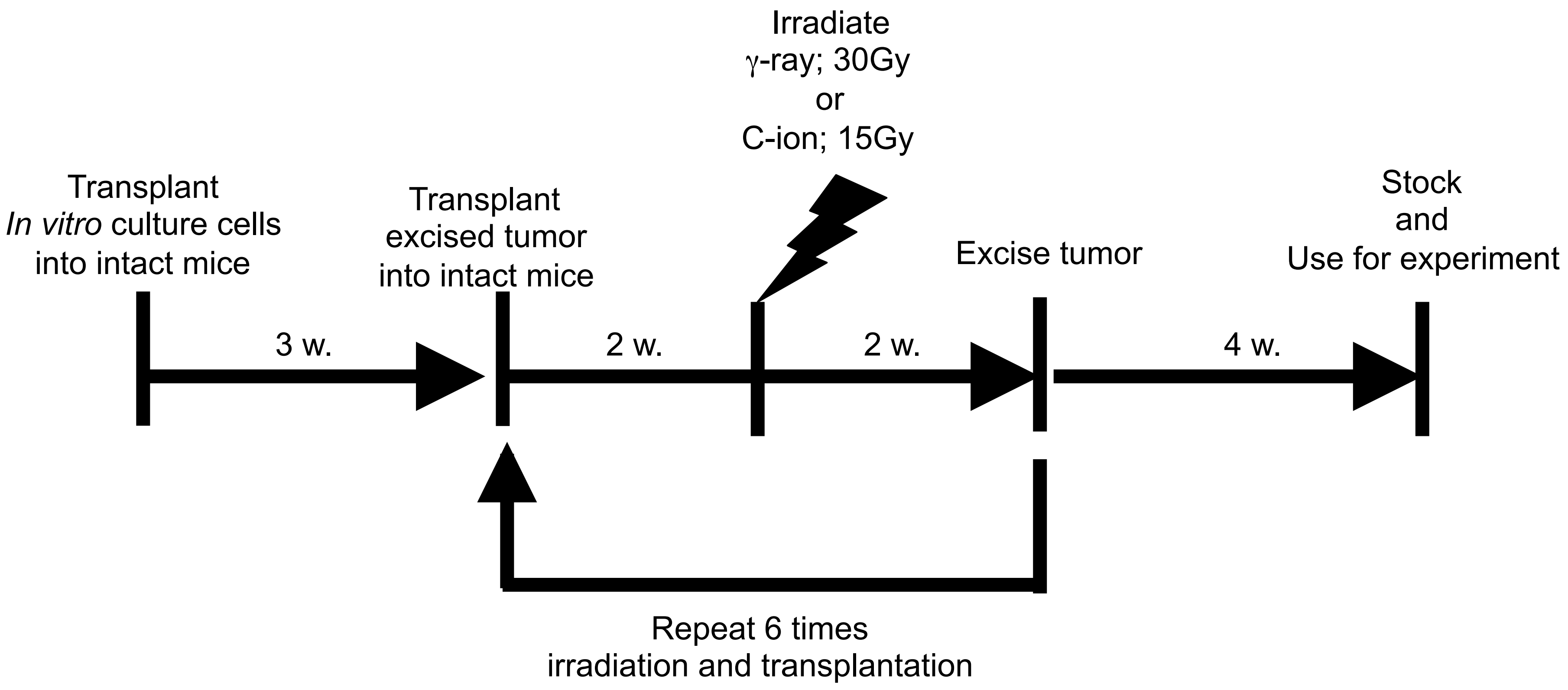

Supplemental figure 2, Shimokawa *et al.*

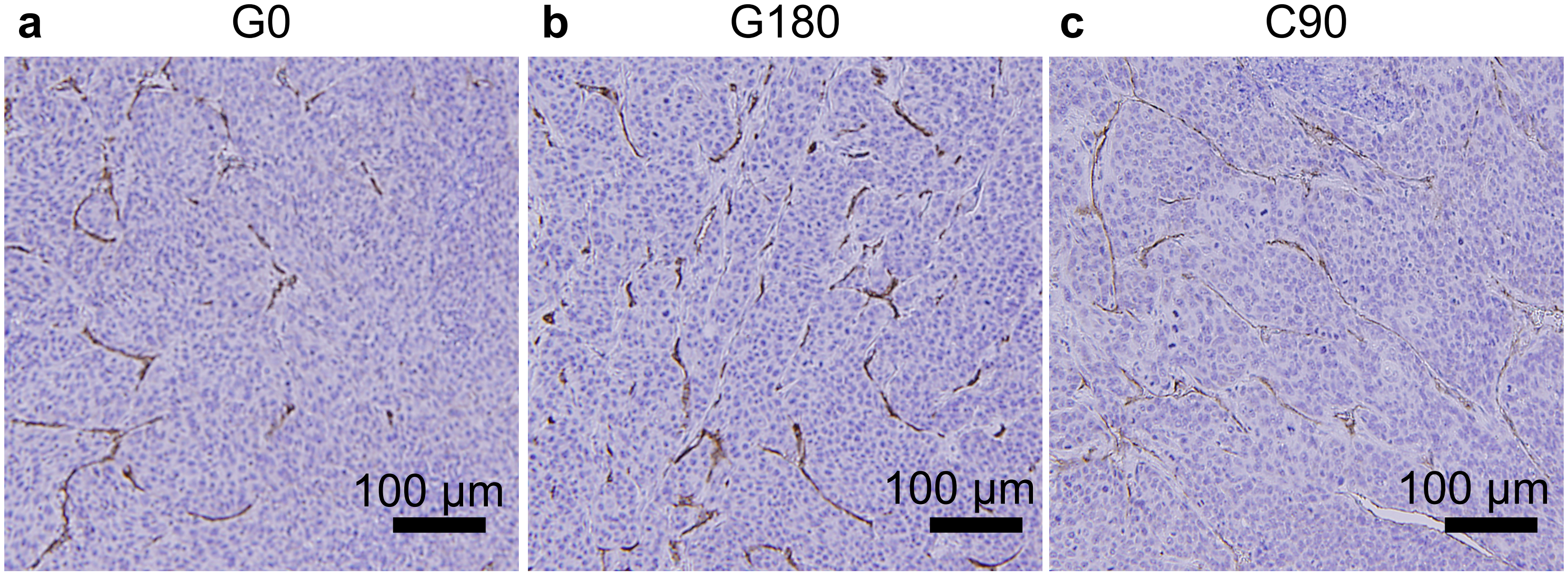

Supplememtal figure 3, Shimokawa *et al*.

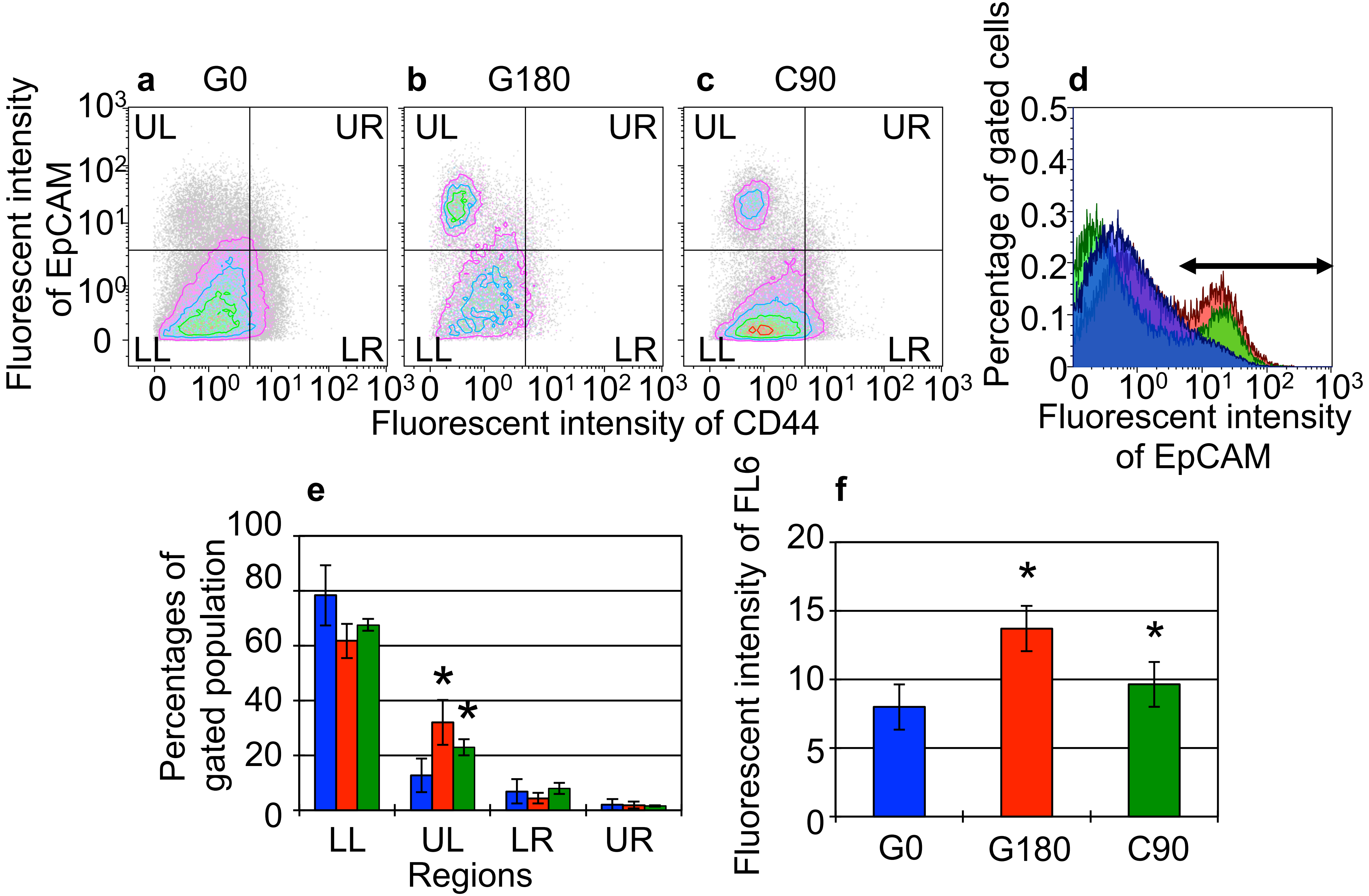

Supplememta figure 4, Shimokawa *et al*.

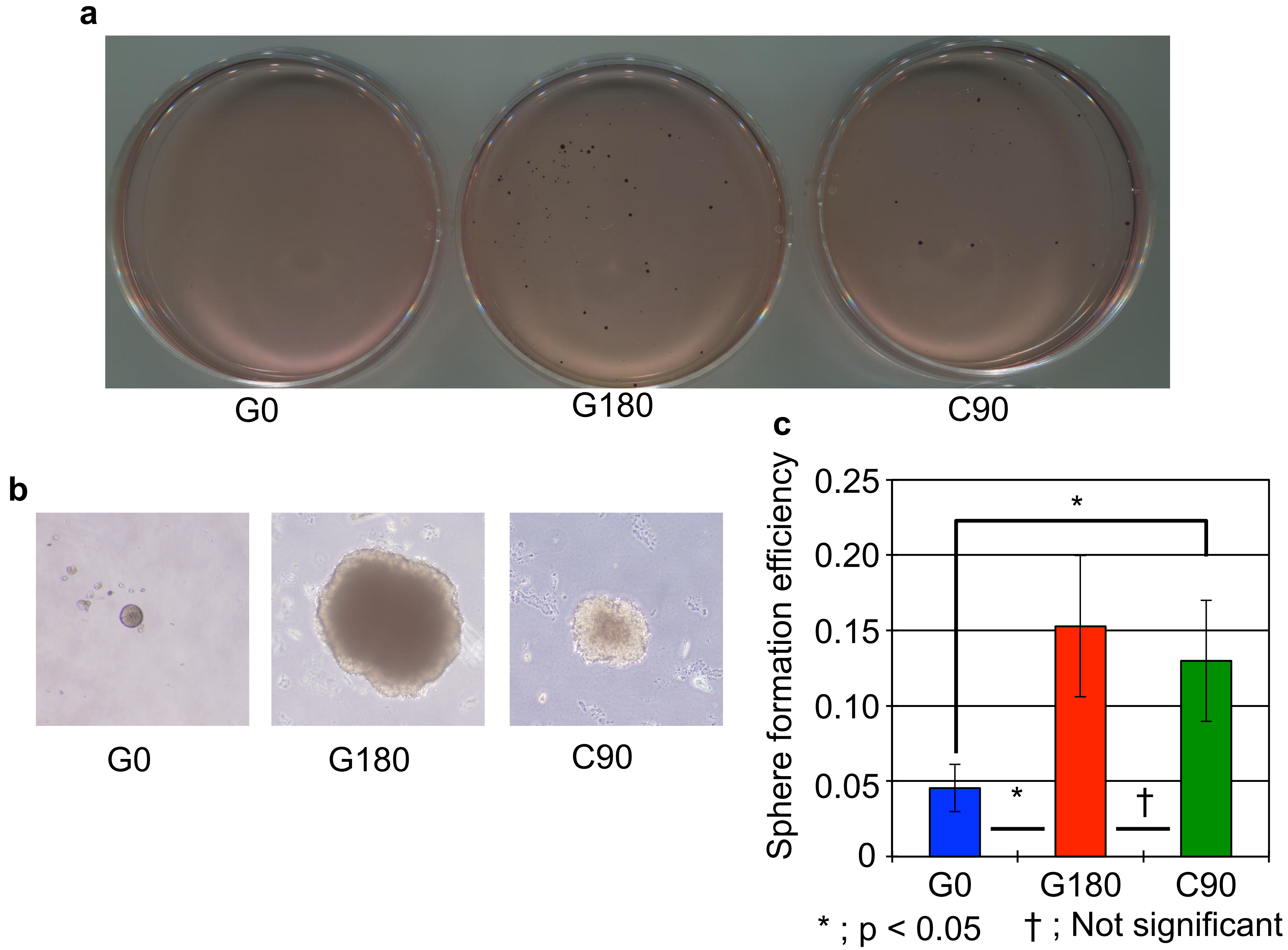

Supplemental figure 5, Shimokawa *et al.*

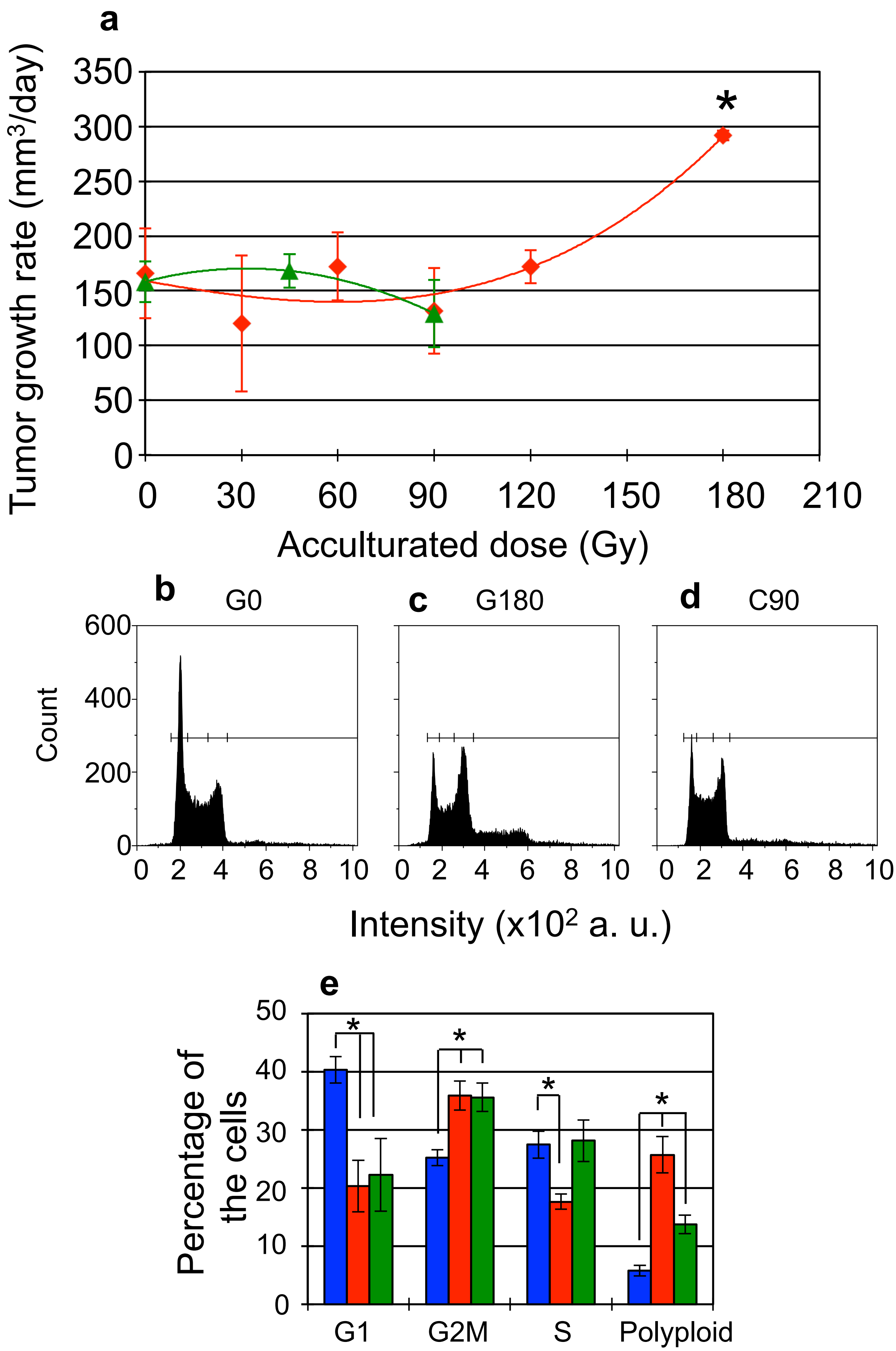

Supplement: Supplementary file 1 — Supplemental information [file 41598_2018_19422_MOESM1_ESM.pdf]
